# The sheep KAP8-2 gene, a new KAP8 family member that is absent in humans

**DOI:** 10.1186/2193-1801-3-528

**Published:** 2014-09-15

**Authors:** Hua Gong, Huitong Zhou, Jolon M Dyer, Jon GH Hickford

**Affiliations:** Gene-Marker Laboratory, Faculty of Agriculture and Life Sciences, Lincoln University, PO Box 84, Lincoln, 7647 New Zealand; Lincoln Research Centre, AgResearch Limited, PO Box 8742, Lincoln, 8140 New Zealand

**Keywords:** KAP8-2 gene (*KRTAP8-2*), PCR-SSCP, Sheep, Variation

## Abstract

The keratin-associated proteins (KAPs) are fundamental components of hair and wool fibres, and are believed to in part be responsible for some of the properties of these fibres. KAPs can be divided into three groups: the high sulphur (HS) KAPs, the ultra-high sulphur (UHS) KAPs and the high glycine-tyrosine (HGT) KAPs. KAP8 is a HGT-KAP family and was believed to be coded for by a single gene in both humans and sheep. However, the recent identification of a KAP8-2 gene in goats led us to investigate whether a KAP8-2 gene exists in sheep.

A BLAST search of the Ovine Genome Assembly v2.0 using the coding sequence of caprine *KRTAP8-2* identified a homologous region on sheep chromosome 1 (OAR1:123005473_123005664; *E* = e^-101^). This region was clustered with a number of previously identified KAP genes including (in order from the centromere) *KRTAP11-1*, *KRTAP7-1, KRTAP8-1, KRTAP6-2, KRTAP6-1*, *KRTAP13-3* and *KRTAP24-1*. PCR-SSCP analysis of the notional gene revealed two dissimilar PCR-SSCP banding patterns, representing two DNA sequences. A single nucleotide difference 21 bp upstream of the TATA box was identified. The two sequences did not have great homology with known ovine *KRTAP* sequences, but high sequence identity was found with *KRTAP8-2* from goats and reindeer. These results suggest that sheep possess a KAP8-2 gene and that this gene is polymorphic.

The notional KAP8-2 protein is comprised of 63 amino acid residues and is rich in glycine and tyrosine, but has a low cysteine content. In contrast to other HGT-KAPs, ovine KAP8-2 contains more acidic amino acid residues, and this would likely result in a lower isoelectric point (pI) of 6.3.

**Electronic supplementary material:**

The online version of this article (doi:10.1186/2193-1801-3-528) contains supplementary material, which is available to authorized users.

## Introduction

The keratin-associated proteins (KAPs) are part of the matrix of wool fibres and form a cross-linked network with the keratin intermediate filaments (Powell & Rogers 1997). They typically possess a high content of either cysteine, or glycine and tyrosine. They can be divided into three broad groups: the high sulphur (HS; ≤30 mol% cysteine) KAPs, the ultra-high sulphur (UHS; >30 mol% cysteine) KAPs and the high glycine-tyrosine (HGT; 35–60 mol% glycine and tyrosine) KAPs (Powell & Rogers 1997).

The HGT-KAPs are largely present in the orthocortex of the wool fibre (Powell & Rogers 1997) and are expressed, shortly after the expression of the keratin intermediate filaments (Rogers 2006). HGT-KAPs vary considerably in abundance both between and within species, ranging from less than 3% in human hair and wool from the Lincoln breed of sheep, through to 4-12% in Merino sheep wool, 18% in the hair of mice and 30-40% in echidna quills (Gillespie 1990). HGT-KAPs are present at a much lower level in the felting lustre wool mutant compared to normal wool (Gillespie & Darskus 1971), and in the felting lustre mutant wool follicles the HGT-KAP genes are down-regulated (Li et al. 2009), suggesting HGT-KAPs have some association with wool crimp.

All the known HGT-KAP genes have been mapped to chromosome 1 in sheep (Gong et al. 2012a) and clustered in a region that harbours a QTL for mean fibre diameter in medium wool Merino sheep (Beh et al. 2001). In a Merino half-sib family, this chromosome region has also been suggested to be associated with variation in wool fibre diameter (Parsons et al. 1994).

In sheep there are type I and type II HGT-KAPs in three families: KAP6 (type I), KAP7 (type II) and KAP8 (type II) (Gong et al. 2012a). KAP6 is a multi-gene family and currently comprises three genes (Gong et al. 2012a; Fratini et al. 1993), whereas KAP7 and KAP8 are currently thought to be single member ‘families’, as only one gene from each family has been identified in sheep (Kuczek & Rogers 1987). The numbers of ovine genes reported in these individual HGT-KAP ‘families’ match well with those identified in the human genome, with reportedly three functional KAP6 genes, one functional KAP7 gene and one functional KAP8 gene (Rogers et al. 2002). Recently, a new KAP8 gene called *KRTAP8-2* was identified in goats (Jin et al. 2011). This suggests that the number of KAP8 genes may vary between species and, given the relatedness of sheep and goats, suggests a second member of KAP8 may exist in sheep.

Here we describe the identification of *KRTAP8-2* in sheep and report genetic variation identified using PCR-SSCP analysis and DNA sequencing.

## Materials and methods

### Sheep and DNA samples

Two hundred and eight New Zealand (NZ) Romney-cross sheep were investigated. The NZ Romney and its crosses are the most common dual-purpose sheep New Zealand and include the Perendale and Coopworth breeds. Samples of blood from these sheep were collected directly onto FTA cards (Whatman BioScience, Middlesex, UK) and DNA for analysis was purified from 1.2 mm punches from the cards, using a procedure described by Zhou et al. ( 2006).

### Bioinformatic analysis of the ovine genome sequence

The coding sequence of the caprine KAP8-2 gene (GenBank AY510123) was used to BLAST search the Ovine Genome Assembly v2.0 (http://www.livestockgenomics.csiro.au/sheep). The sequence that showed the most homology with the caprine sequence was presumed to be the notional ovine KAP8-2 gene.

### PCR primers and PCR amplification

Two PCR primers (5’-taggcagtcagtcatcctg-3’ and 5’-atagagaatatgaagtccacg-3’) were designed based on the sequence homologous to caprine *KAP8-2* identified in the Ovine Genome Assembly v2.0. The primers were synthesized by Integrated DNA Technologies (Coralville, IA, USA).

PCR amplification was undertaken using the purified genomic DNA on one punch of the FTA paper, 0.25 μM of each primer, 150 μM of each dNTP (Bioline, London, UK), 2.5 mM of Mg^2+^, 0.5 U of Taq DNA polymerase (Qiagen, Hilden, Germany) and 1× reaction buffer supplied in a 20-μL reaction. The thermal profile for amplification consisted of 2 min at 94°C, followed by 35 cycles of 30 s at 94°C, 30 s at 60°C and 30 s at 72°C, with a final extension of 5 min at 72°C. This was done in S1000 thermal cyclers (Bio-Rad, Hercules, CA, USA).

Amplicons were visualized by electrophoresis in 1% agarose (Bioline) gels, using 1 × TBE buffer containing 200 ng/mL of ethidium bromide.

### Variant screening and sequencing

PCR amplicons were subject to SSCP analysis. A 0.7-μL aliquot of each amplicon was mixed with 7 μL of loading dye (98% formamide, 10 mM EDTA, 0.025% bromophenol blue, 0.025% xylene-cyanol) and after denaturation at 95°C for 5 min, the samples were cooled rapidly on wet ice and loaded on 16 cm × 18 cm, 14% acrylamide:bisacrylamide (37.5:1) (Bio-Rad) gels. Electrophoresis was performed using Protean II xi cells (Bio-Rad), at 200 V for 18 h at 25°C in 0.5 × TBE buffer. The gels were silver-stained by the method of Byun et al. ( 2009).

PCR amplicons representing individual SSCP patterns were purified using a MinElute PCR Purification kit (Qiagen) and then directly sequenced in both directions.

### Sequence analyses

DNA sequence analyses were carried out using DNAMAN (version 5.2.10, Lynnon BioSoft, Vaudreuil, Canada) and a BLAST search was undertaken of the NCBI GenBank (http://www.ncbi.nlm.nih.gov/) databases using the sequences identified, to find homologous sequences.

## Results

**Figure 1:**
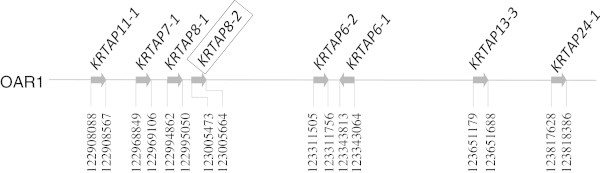
**Location of the putative** ***KRTAP8-2*** **(gene name boxed) together with seven other** ***KRTAPs*** **on sheep chromosome 1.** The coding regions of individual *KRTAPs* are shown, with the nucleotide positions refer to Ovine Genome Assembly v2.0 (http://www.livestockgenomics.csiro.au/sheep). Arrows represent the direction of transcriptions.

**Figure 2:**
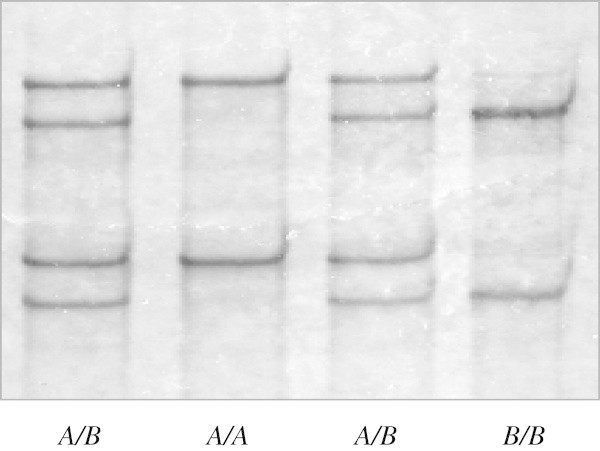
**PCR-SSCP of ovine** ***KRTAP8-2*** **.** Two unique PCR-SSCP patterns representing two variant sequences are shown.

A BLAST search of the Ovine Genome Assembly v2.0 using the caprine *KRTAP8-2* coding sequence (AY510123) revealed a region on sheep chromosome 1 (OAR1:123005473_123005664; *E* = e^-101^) that contained a 192-bp open reading frame and that had 99% homology with the caprine gene. Near this region, seven previously described ovine KAP genes were also identified and these (including *KRTAP8-2*) were *KRTAP11-1*, *KRTAP7-1, KRTAP8-1,****KRTAP8-2****, KRTAP6-2, KRTAP6-1*, *KRTAP13-3* and *KRTAP24-1*(in order from the centromere) (Figure 1). The open reading frame identified had high homology with sheep skin ESTs in GenBank and identical sequences covering the entire open reading frame were found in 44 EST sequences derived from skin tissues (Additional file 1: Table S1).PCR amplification of the entire open reading frame and its flanking sequence generated amplicons with the expected size of 473 bp. SSCP analysis of these amplicons revealed two unique banding patterns, with either one or a combination of two patterns being observed in each sheep (Figure 2).

Sequencing of PCR amplicons revealed that these two PCR-SSCP patterns represented two different DNA sequences. These sequences differed from each other by one nucleotide, 21 bp upstream of the TATA box. Neither of the sequences was identical to the sequence reported in v2.0 of the Ovine Genome Assembly, with three nucleotide differences being detected in the 3’ UT region. This likely reflects either additional genetic variation in the gene, or sequencing/assembly errors within v2.0.

The sheep sequences identified here did not share great homology with any other known ovine *KRTAP* sequence, but sequence similarity was found with the *KRTAP8-2* sequences from goats (AY510123) and reindeer (EF407854), with 99% and 95% similarity respectively in the coding region. These sequences were assumed to represent allelic variants of ovine *KRTAP8-2* and were named variants *A* and *B*. They were placed in GenBank under the accession numbers KF220646 and KF220646, respectively.

Variant *A* was found most frequently (at a frequency of 90.5%), while variant *B* was less common (at a frequency of 9.5%) in the Romney-cross sheep investigated. Three genotypes were observed, with frequencies of 83%, 15% and 2% for *AA*, *AB* and *BB* respectively.

The putative *KRTAP8-2* sequence would encode a 63 amino acid polypeptide, that contained a high level of glycine (23.8 mol%) and tyrosine (20.6 mol%), accounting for 44.4 mol% in total of the amino acid content. It had a moderate amount of phenylalanine (9.5 mol%) and serine (7.9 mol%), but a relatively low cysteine content (3.2 mol%). This polypeptide also possessed 3.2 mol% aspartic acid and 1.6 mol% glutamic acid, amino acids that are absent in other HGT-KAPs. The calculated isoelectric point (pI) of the protein was 6.3 (Table 1).

**Table 1:** **Comparison of the amino acid content (mol%) and pI value of ovine KAP8-2 and other ovine HGT-KAPs**

HGT-KAP	Glycine	Tyrosine	Cysteine	Serine	Phenylalanine	Proline	Aspartic acid	Glutamic acid	pI	Reference
KAP6-1	37.4-37.5	21.7-23.4	9.4-10.8	14.5-15.6	1.6-2.4	0	0	0	8.1-8.3	Gong et al. 2011a
KAP6-2	38.6	21.7	12.1	10.8	2.4	1.2	0	0	8.2	Gong et al. 2011a
KAP7-1	22.4	11.8	5.9	12.9-14.1	10.6	7.1	0	0	8.7	Gong et al. 2012c
KAP8-1	22.6	16.1-17.7	6.5	12.9	9.7	6.5	0	0	8.3	Gong et al. 2012c
KAP8-2	23.8	20.6	3.2	7.9	9.5	6.4	3.2	1.6	6.3	This study

## Discussion

This study has identified a new gene encoding a HGT-KAP in sheep. The gene was grouped with other KAP genes on ovine chromosome 1, but located at a different position and with a lower sequence similarity to these genes. These suggest that this gene represent a previously un-identified ovine KAP gene. The similarity of this gene sequence to the *KRTAP8-2* sequences from goats and reindeer suggests that it is an ovine orthologue of *KRTAP8-2*.

The putative ovine *KRTAP8-2* exhibited sequence variation, with two sequence variants being found. This is consistent with the finding of sequence variation in other ovine *KRTAPs* (Gong et al. 2012a; Gong et al. 2011a; Gong et al. 2011b; Gong et al. 2012b; Gong et al. 2012c; Zhou et al. 2012). However, in contrast to other *KRTAPs*, the variation found in ovine *KRTAP8-2* was not within the coding region, but instead located near the TATA box. This variation may affect RNA polymerase II binding and hence the expression of the gene, but this would need to be confirmed through further investigation.

The predicted ovine KAP8-2 sequence exhibits some characteristics that are consistent with other type II HGT-KAPs, such as the observed high glycine and tyrosine content and higher levels of phenylalanine, but less cysteine (Table 1). However, some unique features are also observed. Firstly, there is a relatively low cysteine content (3.2 mol%), which contrasts with all previously reported KAPs. Secondly the polypeptide contains a high (4.8 mol%) aspartic acid and glutamic acid content. These acidic amino acids are not common in other HGT-KAPs. Lastly it is noteworthy that the polypeptide would likely have a low pI (6.3), as a result of this relatively high level of acidic amino acid residues. Such a low pI value has not been observed in any other HGT-KAP, where the pI is typically higher than 8.

Considering there are two types of keratins that cross-link with the KAPs, and of these the type I keratins are characteristically more acidic (pI 4.5-6.0), while the type II keratins tend to be more basic (pI 6.5-8.5) (Bowden et al. 1987); the predicted lower pI value of KAP8-2 may affect its interaction with keratins, and on a charge basis it would be expected to have a greater affinity for the type II (basic) keratins.

While the protein encoded by the ovine KAP8-2 gene has not yet been isolated from wool, the gene appears to be expressed and functional in sheep as many ESTs with sequences identical to this gene have been reported in skin tissues (Additional file 1: Table S1). A functional orthologue of this gene appears to be absent in humans, a species in which only one functional and two pseudogenic KAP genes are found (Rogers et al. 2002). The KAP8-2 gene is the only KAP gene identified and reported to date that is present in sheep and goats, but is absent in humans. The functional significance of this gene in hair and wool characteristics, and in the evolution of hair and wool, awaits further investigation.

## Electronic supplementary material

Additional file 1: Table S1: Sheep skin ESTs identical to the ovine KAP8-2 gene. (XLS 30 KB)
